# Early Life Vitamin C Deficiency Does Not Alter Morphology of Hippocampal CA1 Pyramidal Neurons or Markers of Synaptic Plasticity in a Guinea Pig Model

**DOI:** 10.3390/nu10060749

**Published:** 2018-06-08

**Authors:** Stine N. Hansen, Jane M. Bjørn Jørgensen, Jens R. Nyengaard, Jens Lykkesfeldt, Pernille Tveden-Nyborg

**Affiliations:** 1 Section for Experimental Animals, Department of Veterinary and Animal Sciences, University of Copenhagen, Ridebanevej 9, 1870 Frederiksberg, Denmark; snoha@sund.ku.dk (S.N.H.); jane_bjoern@hotmail.com (J.M.B.J.); jrnyengaard@clin.au.dk (J.R.N.); jopl@sund.ku.dk (J.L.); 2 Core Center of Molecular Morphology, Section for Stereology and Microscopy, Centre for Stochastic Geometry and Advanced Bioimaging, Department of Clinical Medicine, Aarhus University, Noerrebrogade 44, Building 10G, 3rd Floor, 8000 Aarhus, Denmark

**Keywords:** vitamin C deficiency, cavia porcellus, neuronal morphology, synaptic plasticity

## Abstract

Approximately 15% of the Western world population, including pregnant women and their children, is characterized as vitamin C (vitC) deficient. In guinea pigs, early life vitC deficiency causes spatial memory deficits, decreased hippocampal volume and neuron numbers, in otherwise clinically healthy animals. We hypothesized that vitC deficiency leads to decreased brain-derived neurotrophic factor and synaptic plasticity markers in selected brain areas (frontal cortex, hippocampus and striatum) and cause morphological changes in cornu ammonis 1 pyramidal neurons of the hippocampus either through a direct effect or indirectly by increased oxidative stress. Fifty-seven female guinea pigs were allocated to three groups receiving either 1390, 100 or 0–50 mg vitC/kg feed for 11 weeks. Dietary vitC levels were reflected in the plasma, cortical and adrenal gland levels, however, redox imbalance was only present in the adrenal glands allowing for the investigation of a direct influence of vitC deficiency on the chosen parameters in the brain. Synaptic plasticity markers were not affected in the investigated brain areas and no differences in isolated pyramidal neuron morphology was recorded. Based on our findings, it appears that vitC deficiency may primarily elicit impaired neuronal function through increased levels of oxidative stress.

## 1. Introduction

The developing brain is particularly susceptible to oxidative stress due to a high cellular metabolic activity and immature antioxidant systems [1,2,3]. During this sensitive period, malnutrition has been shown to elicit a negative impact on cognitive function, brain morphology, neuronal development and maturation [4,5,6]. In the Western world, vitamin C (vitC) deficiency is relatively common, with an estimated prevalence of about 15% [7,8,9], including pregnant women and children [10,11,12]. VitC is an essential micronutrient with strong antioxidant properties and the brain displays surprisingly high levels in comparison with other organs [13,14]. During prolonged depletion of dietary vitC, the levels in the brain only decline to approximately 25% of saturated levels, despite close to total exhaustion in most other organs, emphasizing the brain’s unique ability to preserve high vitC levels [15,16]. In addition to being an efficient quencher of free radicals, vitC is also a specific co-factor in several cellular pathways e.g., the recycling of tetrahydrobiopterin (BH4), involved in the synthesis of the monoaminergic neurotransmitters, and acts as a key participant in glutamatergic neurotransmission/glutamate re-uptake [17,18,19]. 

In guinea pigs, early life vitC deficiency increases oxidative stress in the brain resulting in spatial memory deficits, reduced hippocampal volume and neuron numbers, as well as a suggested decrease in neuronal migration in the dentate gyrus [16,20,21,22]. Furthermore, vitC transport to the brain is essential for perinatal survival in mice [23,24], and induced deficiency has been shown to reduce functional performance in the l-gulono-γ-lactone oxidase knockout mice (*Gulo^−/−^*) [25,26]. These studies stress the importance of vitC in the brain and highlight the negative consequences of deficiency during cerebral development; however, the underlying mechanisms behind vitC deficiency promoted deficits in the brain are currently not sufficiently elucidated.

Synaptic plasticity and dendrite development are key neuronal features in memory formation [27,28,29,30]. VitC supplementation to in vitro cultured neurons exposed to a peroxide insult induces brain derived neurotrophic factor (BDNF) [31], while total cessation of vitC supplementation leads to increased oxidative stress and decreased BDNF in the brain in vivo [32]. In the developing brain, BDNF is an important growth factor promoting neuronal survival, dendrite development and synaptic plasticity [33], and plays an essential role in memory formation, including spatial memory [33,34]. Thus, perturbation of the BDNF pathway may be an important contributor to the dysfunctions in spatial memory reported in marginally vitC deficient guinea pigs [20]. This may be by the direct influence of low vitC on BDNF or secondarily through increased oxidative stress. In addition, the active uptake of reduced vitC, ascorbate (Asc) in neurons, achieved through the sodium-dependent vitC transporter 2 (SVCT2), has been shown to be important for neuronal maturation, glutamatergic function, and dendrite morphology and complexity [35,36,37], highlighting another, potentially direct, effect of vitC deficiency on synaptic plasticity, dendrite development and subsequent neuronal signaling. 

**Figure 1:**
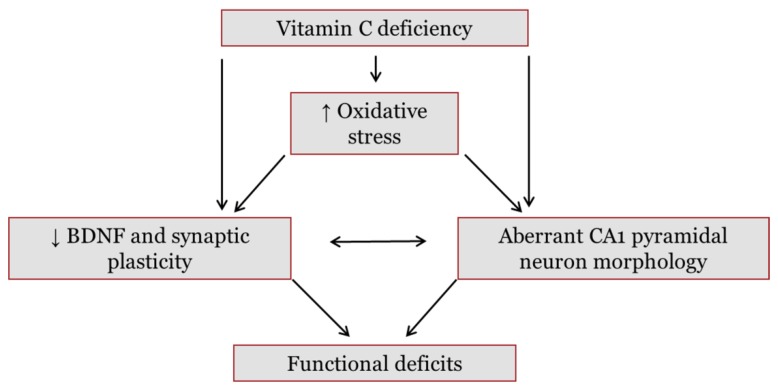
Hypothesized pathways through which vitamin C (vitC) deficiency may cause functional deficits in the brain. Reduced levels of vitC during deficiency may disturb redox balance and subsequently promote oxidative stress in the brain. In addition, vitC deficiency may exert a direct effect on brain function. Both vitC deficiency and increases in oxidative stress have been found to lower levels of brain-derived neurotrophic factor (BDNF) in the brain. A decrease in BDNF, and subsequent negative effects on downstream pathways, may reduce synaptic plasticity. Synaptic plasticity is crucial in memory formation and cognitive functions; hence, dysfunction may lower signal transmission and induce functional deficits. BDNF is also involved in neuronal arbor growth and a reduction may further induce aberrant dendrite development of hippocampal cornu ammonis (CA) 1 pyramidal neurons. These neurons are crucial in memory functions and have been found to be susceptible to increased oxidative stress and states of malnutrition. Collectively, vitC deficiency is hypothesized to cause changes in synaptic plasticity and neuronal morphology, potentially disrupting brain functionality.

The current study investigated the hypothesis that early life vitC deficiency increases oxidative stress and decreases the levels of BDNF in the frontal cortex (FC), the hippocampus (Hip) and the striatum (Stri), which are all regions that are interconnected and involved in spatial memory, motor function, goal-directed behavior and decision making [38,39,40] (Figure 1). Decreased levels of BDNF may decrease protein levels and/or phosphorylation of downstream molecular markers Ca^2+^-calmodulin-dependent kinase II (CAMKII) and synapsin 1, promoting negative effects on synaptic plasticity, dendrite development and neurotransmission [3,41]. Furthermore, vitC deficiency might decrease signal transmission and spatial memory formation by reducing arborization and dendritic spine development of the hippocampal cornu ammonis 1 (CA1) pyramidal neurons, known to be vulnerable to oxidative stress, [42,43,44,45]. Two different degrees of vitC deficiency were compared to controls receiving high levels of vitC, enabling the assessment of a putative dose-related response on the investigated markers.

## 2. Materials and Methods

### 2.1. Animals

All in vivo experiments were approved by the Danish Animal Experiments Inspectorate (License number 2012-15-2934-00205) under the Ministry of Environment and Food and in accordance with EU directive 2010/63/EU and approved by the Animal Care and Use Committee (IACUC, University of Copenhagen, Faculty of Health Sciences) on 11 March 2016 (project number P16-210). All animals were inspected daily by trained personnel and group-housed in floor pens with *ad libitum* access to feed, hay and water. In total, 57 female Dunkin Hartley guinea pigs (Envigo, Venray, The Netherlands) at seven days of age were weight-stratified into three dietary groups (*n* = 19/group) receiving a standard guinea pig maintenance chow (prod. code S9406; ssniff Spezialdiäten GmbH, Soest, Germany) differing only in vitC content. The vitC was added as phosphorylated ascorbate (Stay-C) to extend stability. The total ascorbate content of the feed was subsequently determined by post production analysis. The respective experimental groups received: 1390 mg vitC/kg feed by analysis (Ctrl), 100 mg vitC/kg feed (Def) and 0–50 mg vitC/kg feed (Sev_def) (ssniff Spezialdiäten GmbH, Soest, Germany). The levels of 100 and 50 mg vitC/kg feed were obtained by titration of 123 mg vitC/kg feed (by analysis) with 0 mg vitC/kg feed. The Sev_def group was kept on 0 mg vitC/kg feed for 16 days, until the first animals showed signs of weight stagnation (a pre-scorbutic clinical sign) after which the entire group was immediately transferred to 50 mg vitC/kg feed. Three Sev_def animals received an oral dose of softened vitC containing feed on day 16 and 17 to alleviate pre-scorbutic symptoms. The vitC levels of the Ctrl and Def diets were based on previously published data, ensuring vitC levels in Ctrl and low, albeit non-scorbutic levels in Def animals [13,20,21]. The Sev_def group was included in the study to increase effects of low vitC by first inducing a severe, borderline scorbutic vitC deficiency through total dietary depletion and subsequently keeping the animals on a low but non-scorbutic vitC containing diet. All animals were weight-monitored weekly, except for Sev_def animals, which were weighed every other day during the first 16 days. Apart from the three Sev_def animals which were briefly treated for symptoms of scurvy to a full recovery, no other clinical signs of decreased animal welfare were recorded during the study period. 

After 11 weeks on the diet, the animals were sedated with 0.2 mL/kg butorphanol (Torbugesic, Scanvet, Fredensborg, Denmark) and anaesthetized with 3–5% isoflurane (Isoba Vet, Intervet International, Boxmeer, The Netherlands), until cessation of voluntary reflexes after which an intra-cardiac blood sample was obtained and plasma subsequently collected as previously described [46]. The animals were euthanized by decapitation and the brains were removed, rinsed in ice-cold PBS, weighed and divided into hemispheres. The right hemispheres from 27 randomly chosen animals were allocated to another study. For the remaining 30 animals, the right hemispheres from Ctrl and Sev_def were preserved for the Golgi-analysis, while the right hemispheres from the Def group were not included in this part of the study. The left hemispheres from all animals in the three groups were divided into FC, Hip, Stri (defined by anatomical fix-points with reference to the rat brain [47]) and the residual cerebral cortex, and immediately snap-frozen in liquid nitrogen. The adrenal glands were removed, rinsed in PBS; the left was snap-frozen in liquid nitrogen and the right fixated in 4% paraformaldehyde for future analysis.

### 2.2. Biochemistry

Plasma levels of total vitC, dehydroascorbate (DHA), malondialdehyde (MDA), α- and γ-tocopherol, BH4 and dihydrobiopterin (BH2) were measured. In tissue samples, the residual cerebral cortex and adrenal gland, analyses of vitC, DHA, glutathione (GSH), glutathione disulfide (GSSG), superoxide dismutase (SOD), MDA (not adrenal glands), α- and γ-tocopherol were conducted. Both plasma and tissue samples were analyzed as previously described [22,46,48,49,50]. 

Briefly, plasma samples for total vitC and DHA were stabilized 1:2 with 10% meta-phosphoric acid and analyzed by electrochemical detection on HPLC. DHA% was calculated as the percentage of total vitC, which constitutes DHA. MDA was measured by fluorometric detection on HPLC. BH4 was stabilized with 4% dithioerythritol and quantified by fluorometric detection on HPLC. For tissue samples the tissue was homogenized 1:10 in PBS and for total vitC, DHA, GSH and GSSG, the samples were stabilized 1:2 with 10% meta-phosphoric acid. GSH and GSSG were measured using spectrofluorometry and vitC and DHA by electrochemical detection on HPLC. For SOD, a Ransod kit was used. α- and γ-tocopherol were measured by HPLC using electrochemical detection.

### 2.3. Protein Extraction

The protein extraction was performed on ten randomly chosen animals from each group as previously described [51]. Briefly, 40 mg of tissue from each of FC, Hip and Stri was dissected on ice. The tissue samples were homogenized using a Potter–Elvehjem in 500 μL RIPA buffer (50 mmol/L tris pH 8.0, 150 mmol/L sodium chloride, 1% Triton X-100, 0.5% sodium deoxycholate and 0.1% sodium dodecyl sulfate) with 1:100 protease inhibitor cocktail (Sigma-Aldrich, Darmstadt, Germany) and 1:100 phosphatase inhibitor cocktail (Sigma-Aldrich, Darmstadt, Germany). The samples were centrifuged for 10 min at 12,000 rpm at 4 °C and the protein extract was stored at −80 °C in aliquots. Protein concentration was determined by a commercial BCA kit according to manufacturer’s instructions (Merck Millipore, Darmstadt, Germany).

### 2.4. ELISA

BDNF levels were analyzed in FC, Hip and Stri using a commercial ELISA kit (SEA011Gu, Cloud-Clone Corp, Houston, TX, USA) as instructed by the manufacturer. All samples were diluted 1:20 in sterile PBS as determined by a dilution series to obtain an optical density close to the center of the linear part of the standard curve. All results were normalized to protein concentration. Four animals across all three groups had to be excluded from the FC analysis and three from the Hip for technical reasons.

### 2.5. Western Blotting

**Figure 2:**
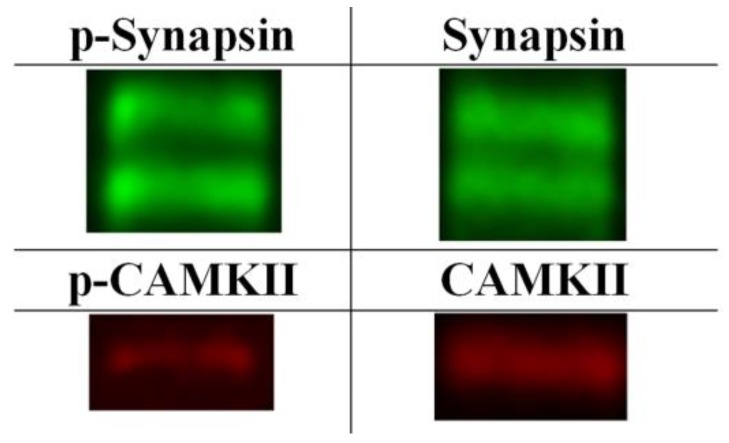
Examples of Western blots of *p*-/synapsin 1 and *p*-/Ca^2+^-calmodulin-dependent kinase II. The figure depicts examples of the Western blots of the four synaptic plasticity markers investigated. The two bands seen in the *p*-synapsin and synapsin Western blots (70 and 74 kDa respectively) are consistent with splice variants as confirmed by manufacturer. P-CAMKII and CAMKII were detected around 50 kDa in accordance with previous findings.

Ten µg of protein from each of FC, Hip and Stri were adjusted to a volume of 11.25 µL, before adding 3.75 µL Laemmli buffer (Bio Rad, Hercules, CA, USA) with 1:10 mecaptoethanol (Sigma-Aldrich, Darmstadt, Germany). The samples were denatured at 70 °C for 10 min before transfer to a 7.5% Criterion™ TGX™ Precast Midi Protein Gel, 26 well, 15 µL/well (Bio Rad, Hercules, CA, USA) and the electrophoresis was run for approximately 40 min. Afterwards the proteins were transferred to a PVDF membrane [51]. All samples were run in duplicates and normalized to total protein levels (REVERT™ Total Protein Stain, Li-Cor, Lincoln, NE, USA). The following antibodies were applied: Anti-synapsin 1 (ab8, Abcam, Cambridge, United Kingdom; 1:1000); *anti*-CAMKII (Cba-2, Thermo Scientific, Waltham, MA, USA; 1:4000); anti-phosphorylated CAMKII (*p*-CAMKII) (22B1, Thermo Scientific, Waltham, MA, USA; 1:1000); anti-phosphorylated synapsin 1 (*p*-synapsin 1) (NB300-181, Novus Biologicals, Littleton, CO, USA; 1:1000). All primary antibodies were incubated at 4 °C o/n. As secondary IRDye^®^ 800CW Donkey-anti-Rabbit IgG (Li-Cor, Lincoln, NE, USA; 1:15,000) and IRDye^®^ 680RD Donkey-anti-Mouse IgG (Li-Cor, Lincoln, NE, USA; 1:15,000) were applied for one hour at RT. The analyses were done using Image Studio 5.2 (Li-Cor, Lincoln, NE, USA) by an observer blinded to the experimental group. Examples of Western blots positive for the selected markers, are provided in Figure 2.

### 2.6. Golgi-Staining and Section Processing

Only Ctrl and Sev_def animals were used for the Golgi-staining and 3D reconstruction of neurons. To Golgi-stain the right hemispheres, a commercial kit was purchased (FD Rapid GolgiStain™, FD NeuroTechnologies, Inc., Colombia, MD, USA) and the staining performed as per manufacturer’s instructions [52]. In summary: The hemispheres were stored in A + B solution for 14 days with a solution change after 24 h and then transferred to C solution for seven days with a solution change after 24 h. The hemispheres were then snap-frozen in isopropanol and stored at −80 °C until further processing.

The hemispheres were mounted on a HM 569 M Cryostat (Mikrom, Walldorf, Germany) and the Hip was cut into 200 μm coronal sections in its entirety. The sections were mounted on 2% gelatin-coated slides and left to dry for one hour at RT after which they were rinsed in miliQ water and placed in D + E solution for 10 min, before being dehydrated in alcohol, cleared in xylene and cover slipped.

### 2.7. Image Acquisition and Analysis

Z-stacks of neurons with somas lying within the stratum pyramidale of the CA1 area of the Hip were captured with newCAST, VIS (Visiopharm, Hoersholm, Denmark) using Olympus BX50F microscope (Olympus Optical co., LTD., Tokyo, Japan) and 60X oil objective (PlanApo, NA 1.40, Oil, Olympus, Japan) applying a step size of 1 μm [52,53]. Neurons were preferentially selected with the soma situated in the middle of the z-axis of the section (between 80 and 150 μm post-dehydration). To reconstruct and analyze the neurons the automatic filament tracer in Imaris (Bitplane, Zürich, Switzerland) was applied. From each animal, five to seven apical and basal dendritic trees, respectively, were analyzed and averaged by an observer blinded to the experimental group [52,53]. The Sholl analysis was conducted in the program by concentric circles with a radius difference of 20 μm radiating from the center of the soma [52]. An overview of the measured morphological parameters and the expected outcome of vitC deficiency is shown in Table 1.

### 2.8. Statistical Analysis

The data was analyzed in GraphPad Prism 7 (GraphPad Software, La Jolla, CA, USA) either by repeated-measures two-way ANOVA, one-way ANOVA or Student’s *t*-test. Equality of variances was assessed by the Brown-Forsythe test, while normal distribution was assessed by the D’Agostino & Pearson normality test and histograms. In the event of nonhomogeneous variances, the data was log-transformed or a nonparametric test was applied. Tukey’s or Dunn’s multiple comparisons test were applied to correct for multiple comparisons in the biochemical and Western blotting data. Results are presented as mean ± SD, or median (25%, 75% quartiles). A *p*-value < 0.05 was considered statistically significant. As DHA% is calculated as (total vitC − Asc)/total vitC × 100 [48], DHA% may—when close to zero—occasionally give rise to negative values. In this study, negative values have been set to zero, while statistics have been performed on the original data to preserve normal distribution and variance.

## 3. Results

### 3.1. Animals

**Figure 3:**
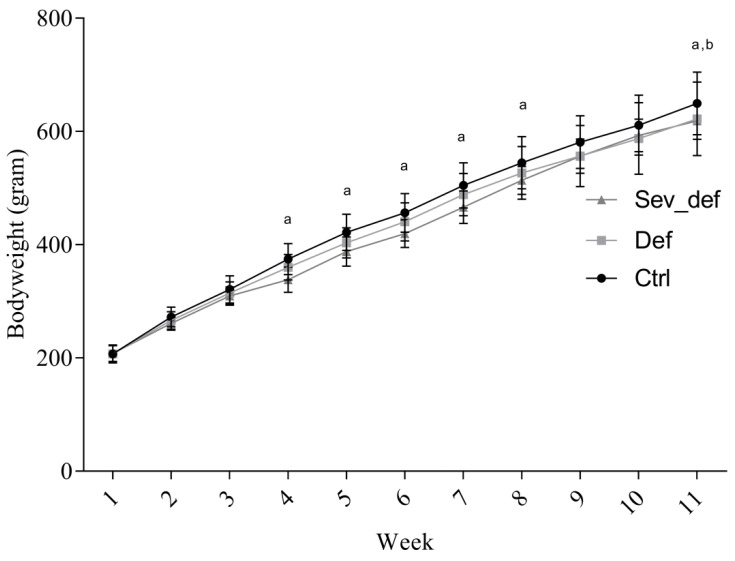
Changes in guinea pig bodyweight during the study. The Sev_def group showed mild growth retardation from week 4, but caught up with Def animals by week 9. At termination, there was a significant difference between Ctrl and Def and Sev_def. ^a^ Ctrl vs. Sev_def, *p* < 0.05; ^b^ Ctrl vs. Def, *p* < 0.05. Two-way repeated-measures ANOVA with Tukey’s multiple comparison correction, *n* = 19, mean ± SD. Ctrl: Control animals, Def: Vitamin C deficient animals, Sev_def: Severely vitamin C deficient animals.

There was a significant impact of time and diet on the bodyweight (time *p* < 0.0001; diet *p* = 0.0436; interaction *p* < 0.0001). The Sev_def group displayed mild growth retardation, when compared with Ctrl from week 4 to week 8 (week 4, *p* = 0.0038; week 5, *p* = 0.0078; week 6, *p* = 0.0036; week 7, *p* = 0.0021; week 8, *p* = 0.0170) but caught up in week 9 and 10 (Figure 3). At study termination, there was a significant difference in the bodyweight of both Def and Sev_def compared to Ctrl (Def *p* = 0.0416; Sev_def *p* = 0.0170). 

### 3.2. Biochemistry

As expected, the vitC levels in plasma reflected the respective diets of the groups (*p* < 0.0001): the Def and Sev_def groups had significantly lower plasma levels than the Ctrl (*p* < 0.0001) (Table 2) at euthanasia. The two deficient groups were also significantly different from each other (Def vs. Sev_def *p* < 0.0001). BH4 concentration in plasma was significantly higher in Ctrl than Def and Sev_def (*p* < 0.0001), while Sev_def was significantly lower than Def (Def vs. Sev_def *p* = 0.031) (Table 2). This was also reflected in the BH2/BH4 ratio, which was significantly lower in Ctrl animals compared to Def and Sev_def (*p* < 0.0001), while there was no difference between the two deficiency groups. There were no differences between the groups in plasma α- and γ-tocopherol and MDA (Table 2).

Dietary vitC intakes were also reflected in the vitC levels in the cerebral cortex: Sev_def levels were lower than Def (*p* < 0.0001) and both Sev_def and Def levels were lower than Ctrl (*p* < 0.0001) (Table 2). Despite reaching significant differences between groups (*p* < 0.0001), DHA% was consistently very low and likely negligible (Ctrl vs. Sev_def *p* < 0.0001; Def vs. Sev_def *p* = 0.0413). No other markers showed any differences between the groups (Table 2).

In the adrenal glands, total vitC in Def and Sev_def were significantly lower (*p* < 0.0001), when compared with Ctrl, but there was no difference between Def and Sev_def groups (Table 2). The DHA% was significantly increased in Sev_def, when compared with Ctrl (*p* = 0.0038), while GSH was increased in both Def and Sev_def (*p* = 0.0220 and *p* = 0.0003, respectively) (Table 2). No other markers showed any differences between the groups.

### 3.3. ELISA and Western Blotting

**Figure 4:**
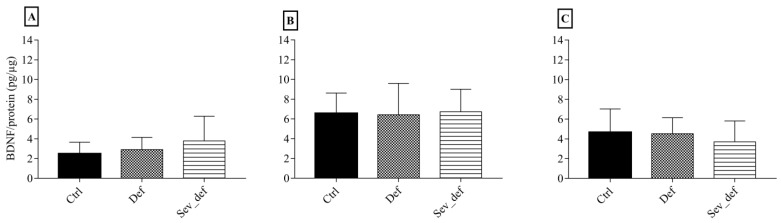
Brain-derived neurotrophic factor levels in the frontal cortex, hippocampus and striatum. Frontal cortex (**A**), the hippocampus (**B**) and the striatum (**C**) as measured by ELISA. No differences between groups were detected in any of the brain areas. One-wayANOVA with Tukey’s multiple comparison test, *n* = 6–10, mean ± SD. Ctrl: Control animals, Def: Vitamin C deficient animals, Sev_def: Severely vitamin C deficient animals.

The ELISA results of BDNF levels in FC, Hip and Stri are shown in Figure 4. No influence of dietary vitC on BDNF was detected in any of the three brain areas. The same was evident for the Western blotting results of synaptic plasticity markers: *p*- and synapsin 1, *p*- and CAMKII and their respective ratios, where no differences due to vitC deficiency could be detected (Table 3). 

### 3.4. Morphological Data

**Figure 5:**
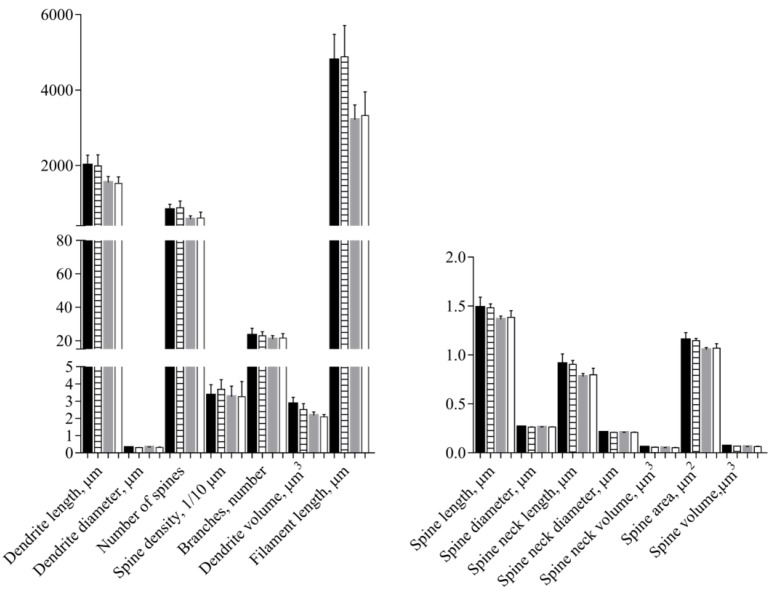
Morphological analyses of apical and basal dendrites of pyramidal neurons in the cornu *ammonis* 1 of the hippocampus. Control animals (apical—black; basal—gray) and severely vitamin C deficient animals (apical—horisontal stripes; basal—white). No influence of vitamin C deficiency was detected in the morphological parameters, except for a slight decrease in apical dendrite volume (*p* = 0.0488). *t*-Test or Welch’s *t*-test, mean ± SD. Each experimental group consisted of 10 animals and from each animal 5–7 pyramidal neurons situated within the stratum pyramidale of cornu ammonis 1 were analyzed for morphological changes and the data averaged.

**Figure 6:**
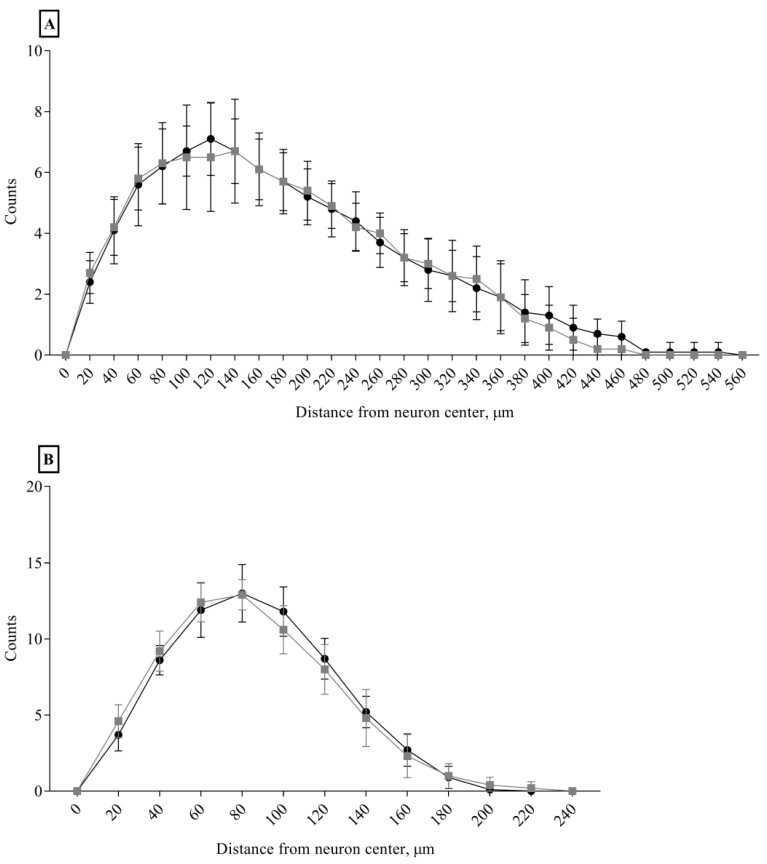
Sholl analysis of apical and basal dendrites of cornu ammonis 1 pyramidal neurons. Apical (**A**) and basal (**B**) cornu ammonis 1 pyramidal neuron from control animals (black dots) and severely vitamin C deficient animals (gray squares). No effect of vitC deficiency was detected on the dendrite complexity measured as number of dendrite crossings on concentric circles radiating from the neuronal soma with a continuous increase in the radius of 20 μm. Two-way repeated measures ANOVA, mean ± SD. Each experimental group consisted of 10 animals and from each animal 5–7 pyramidal neurons within the stratum pyramidale of cornu ammonis 1 were analyzed for morphological changes and the data averaged.

No differences between the Ctrl and Sev_def groups were detected in any of the morphological parameters of the apical and the basal dendrites (Figure 5, Table 1), except for a slight decrease in apical dendrite volume in the Sev_def group (*p* = 0.0488). The same was also the case for the Sholl analysis (Figure 6), where severe vitC deficiency did not give rise to any regional changes in the dendrite arbor for basal and apical dendrites.

## 4. Discussion

The dietary vitC levels were reflected in the recorded plasma concentrations and cortical levels of the animals, as expected. A decrease in BH4 and an increase in BH2/BH4 ratio were evident in plasma of vitC deficient animals, reflecting that Asc is also a cofactor for the recycling of BH4 [17,50]. Though tissue BH4 levels were not measured in this study, the decreased BH4 in plasma may reflect a decrease in cerebral BH4 levels. Inside the brain, BH4 is involved in the synthesis of the monoaminergic neurotransmitters, e.g., dopamine and norepinephrine [19]. Dopaminergic neurons and receptors have been shown to be involved in spatial memory [56,57], while norepinephrine has been found to be involved with memory consolidation and retrieval in the Hip [58,59]. Deviations in the hippocampal monoamine levels could be a likely contributor to vitC imposed cognitive deficits. Indeed, monoaminergic changes have been reported in a knockout mouse model of vitC deficiency [26,60]. 

Only limited changes in additional biochemical markers of redox balance were recorded. The Sev_def group was included to assess effects of an increased degree of vitC deficiency and subsequent progressive negative effects compared to Def animals, possibly indicating a dose-dependency in the recorded markers. Dietary regimes similar to those applied in this study have previously been shown to increase DHA%, as well as other markers of oxidative stress in guinea pigs [20,21,22]. Contrary to our expectations, and despite a decrease in total vitC levels in the cerebral cortex and an increase in the adrenal DHA% of the Def and Sev_def groups, the diets of the current study did not impose a degree of deficiency in the brain severe enough to affect the additional redox markers at time of euthanasia. Why this is the case remains speculative but may be due to the genetic variation of the animals, as vitC homeostasis, and possibly also susceptibility to oxidative stress is influenced by genetic differences [61,62,63]. Thus, it can be surmised that the animals in this study were less susceptible to oxidative stress induced by low dietary vitC compared to previous studies. Still, as vitC has several specific functions, low tissue levels could potentially affect the brain negatively. The present absence of oxidative stress consequently allowed us to investigate a direct influence of vitC deficiency on the BDNF pathway and the morphology of the CA1 pyramidal neurons.

However, since no differences between the experimental groups could be recorded in the markers of synaptic plasticity, no direct impact of decreased vitC on synaptic plasticity can be supported by this study. Consulting previously published findings, it is likely that oxidative stress is imperative for vitC deficiency induced deficits [32,64]. Whether other cellular pathways are directly affected by vitC deficiency remains to be investigated. However, BDNF affects down-stream targets through three alternate routes; the phospholipase C-γ, the phosphoinositide-3 kinase and the extracellular signal-regulated kinase (ERK) regulated pathway; all leading to transcription factor activation [33]. In vitro, Asc and SVCT2 overexpression have been shown to increase ERK 1/2 phosphorylation [37] known to be important for long term spatial memory formation [65] and sensitive to oxidative stress [66]. Synapsin 1 has several phosphorylation sites, including one activated by ERK 1/2 [67]. Despite an absence of changes in synapsin 1 phosphorylated at the CAMKII site as investigated in the current study [68], it may be speculated that vitC deficiency would decrease ERK1/2 phosphorylation, in turn decreasing presynaptic synapsin 1 phosphorylation and/or impaired postsynaptic transcription and leading to the previously observed decreased spatial memory retention in early life guinea pigs [20]. 

Except for a slight decrease in apical dendrite volume, CA1 pyramidal neuron morphology was not significantly different in severe vitC deficient animals compared to control counterparts. This contrasts with previous studies investigating vitC and neuronal morphology. In vitro, cultures of hippocampal neurons from SVCT2 knockout mice display decreased dendrite length and number of primary branches [36], while overexpression of the transporter increased branching in neuroblastoma cells [37], supporting that neuronal morphology, in vitro, is influenced by vitC uptake. In vivo, findings from adult *Gulo^−/−^* mice further associates total vitC depletion with atrophic neuronal changes, including axonal and dendritic shrinkage in the granule and Purkinje neurons of the cerebellum, in conjunction with increases in oxidative stress, apoptotic markers and inflammation [32]. Other forms of malnutrition, both pre-and postnatal, have been found to alter neuronal morphology in the Hip, supporting that the hippocampal dendritic arbor is sensitive to malnutrition states during development; e.g., iron deficiency inducing reduced CA1 pyramidal neurons dendrite width, spine head diameter and branching, [69], a high fat diet giving rise to decreased dendrite length and branch points as well as indications of decreased spines in basal CA1 pyramidal neurons [70], and iodine deficiency showing decreases in the length and number of basal dendrites [71]. This links malnutrition to deviations in early life development of neuronal and dendrite morphology, promoting neurological deficits with putatively negative consequences for brain function i.e., memory deficits [72,73] We hypothesized that vitC deficiency in early life would impose a detrimental effect on overall neuronal morphology, measured as a reduction in dendrite length and branching and an immature or degenerative dendritic spine morphology with alterations in length and reductions in volume and/or area (Table 1) [54,55]. 

Contrary to our hypothesis, but in line with the protein expression analysis on the included brain samples, no alterations of the measured morphological target points were recorded in the current study. Decreased spine density and dendrite length in CA1 has been shown in induced brain-oxidative stress in vivo [74,75] and in animal models of Alzheimer’s disease [76,77], known to display increased oxidative stress during disease progression [78], supporting the association between oxidative stress and impaired CA1 morphology. The absence of morphological differences between experimental groups in the current groups, suggests that vitC deficiency in the brain may require the presence of redox imbalance to impose negative effects on neuronal morphology. 

Other subareas and cell types in the Hip are involved in spatial memory and may also be targets of its deficiency-associated memory deficits. The CA1 area selected for the present investigation represents a subdivision of the Hip and the reported absence of altered neuronal morphology does not exclude a vitC deficiency-imposed effect on other areas of the Hip, e.g., the CA3 and dentate gyrus. CA3 pyramidal neurons are involved in retention of spatial memory and in spatial memory recall [79,80], while neurogenesis in the dentate gyrus has also been found to be crucial in spatial memory [81,82]. Whether other hippocampal areas are affected by vitC deficiency remains to be explored. Other cell types within the brain may also be sensitive to vitC deficiency. Astrocytes are key cells in regulation of oxidative stress in the brain and recycle Asc from DHA [83]. They have been shown to influence neurotransmission, synaptic plasticity, learning and memory [84,85] and increased oxidative stress has been found to induce astrocyte dysfunction [86,87]. Astrocytes secrete Asc, when stimulated by glutamate [88], which may be crucial in preventing excitotoxicity otherwise leading to increased neuronal death [36]. Interestingly, increased oxidative stress decreases accumulation of recycled Asc in astrocytes [89], which may lead to increased glutamate-induced excitotoxicity. Indeed, we have previously found lower number of neurons in the Hip in vitC deficient guinea pigs [20]. Thus, disturbances in astrocyte function and impairment of glutamate regulation could be detrimental to the brain and should be investigated further. 

## 5. Conclusions

In conclusion, vitC deficiency without ensuing oxidative stress does not seem to decrease synaptic plasticity or CA1 neuronal morphology in young guinea pigs. Other pathways potentially involved with vitC deficiency induced spatial memory deficits should be investigated.

## Figures and Tables

**Table 1:** Parameters included in the morphological analysis of cornu ammonis 1 pyramidal neurons. Vitamin C deficiency was expected to cause aberrant neuron morphology either directly or through subsequent oxidative stress thereby decreasing dendrite branching and length of cornu ammonis 1 pyramidal neurons causing an overall decrease in filament length. Furthermore, deficiency induced spine degeneration was expected to reduce total spine numbers and spine density, while also affecting the morphology of the spines. The change in spine morphology inflicted by deficiency was hypothesized to cause a shift towards a more immature/degenerated state of the dendritic spines with decreases in volume, area, diameter and shortening of the neck. Spine length could be increased due to an increase of immature filopodia or decreased due to degeneration [54,55]. The descriptions of the morphological parameters are based on descriptions in the Imaris manual provided by Bitplane.

Parameter	Explanation	Expected Outcome of Vitamin C Deficiency
Dendrite length	The sum of the lengths of all dendrites on a neuron	↓
Dendrite diameter	The mean diameter within a dendrite	↔ or ↓
Dendrite volume	The volume of a dendrite	↔ or ↓
Branch numbers	The number of dendrite branches on an neuron	↓
Spine number	The number of spines on the entire neuron	↓
Spine density	The number of spines per 10 μm dendrite	↓
Filament length	The sum of dendrite length and spine length	↓
Spine length	The length of the spines	↑ or ↓
Spine diameter	The mean of the spine diameter	↓
Spine area	The surface area of the spine	↓
Spine volume	The volume of the spine	↓
Spine neck length	The length of the spine neck	↓
Spine neck diameter	The diameter of the spine neck	↓
Spine neck volume	The volume of the spine neck	↓

**Table 2:** Biochemical parameters of plasma, residual cortex and the adrenal glands.

	Group/Marker	Ctrl (*n* = 19)	Def (*n* = 19)	Sev_Def (*n* = 19)	One-Way ANOVA or Kruskal-Wallis Test
Plasma	VitC (μM) *	43.3 ± 8.5 ^a^	4.4 ± 0.91 ^b^	2.2 ± 0.46 ^c^	*p* < 0.0001
	DHA (%) ^#^	1.9 ± 2.0	3.0 ± 3.5	2.8 ± 3.5	NS
	BH4 (nM)	68.3 ± 8.3 ^a^	47.5 ± 10.1 ^b^	40.4 ± 6.4 ^c^	*p* < 0.0001
	BH2/BH4 ^¤^	0.18(0.16; 0.22) ^a^	0.31(0.25; 0.43) ^b^	0.36(0.33; 0.41) ^b^	*p* < 0.0001
	α-tocopherol (μM)	2.7 ± 0.8	2.5 ± 0.7	2.5 ± 0.7	NS
	γ-tocopherol (μM)	0.1 ± 0.06	0.1 ± 0.05	0.09 ± 0.04	NS
	MDA(μM)	1.0 ± 0.1	1.1 ± 0.2	1.1 ± 0.2	NS
Residual cerebralcortex	VitC (nmol/g)	1320 ± 254.6 ^a^	753.2 ± 70.7 ^b^	393.6 ± 91.3 ^c^	*p* < 0.0001
	DHA (%) ^¤ #^	2.2(1.6; 2.6)	1.6(0.1; 1.9) ^b^	0.0(0; 0.3) ^c^	*p* < 0.0001
	GSH (nmol/g)	1244 ± 113.7	1247 ± 79.0	1244 ± 76.4	NS
	GSSG (%)	3.7 ± 1.9	4.0 ± 1.8	3.9 ± 1.1	NS
	SOD (U/g)	235 ± 54.0	232 ± 63.3	229 ± 79.9	NS
	MDA (nmol/g) ^¤^	165.9(135.1; 200.1)	169.5(140.7; 203.6)	200.8(152.5; 254.9)	NS
	α-tocopherol (nmol/g)	12.6 ± 3.1	11.9 ± 3.3	11.9 ± 1.6	NS
	γ-tocopherol (nmol/g)	0.6 ± 0.2	0.6 ± 0.2	0.6 ± 0.2	NS
Adrenal glands	VitC (nmol/g)	9571 ± 1951 ^a^	3353 ± 1606 ^b^	2554 ± 2344 ^b^	*p* < 0.0001
	DHA (%)	3.5 ± 1.9 ^a^	4.8 ± 2.0 ^a b^	5.7 ± 2.1 ^b^	*p* = 0.0052
	GSH (nmol/g)	1531 ± 107 ^a^	1670 ± 172 ^b^	1745 ± 180 ^b^	*p* = 0.0004
	GSSG (%)	2.1 ± 1.5	1.8 ± 1.5	2.1 ± 1.7	NS
	SOD (U/g)	180 ± 55.6	158 ± 52.0	173 ± 57.3	NS

Despite a clear influence of diet on vitamin C levels in plasma, cortex and adrenal glands, only the adrenal glands showed sign of increased oxidative stress. One-way ANOVA or Kruskal–Wallis with Tukey’s or Dunn’s multiple comparison tests, *n* = 19, mean ± SD for parametric analysis, if non-parameteric: median (25%; 75% quartiles) are reported. * Denotes log transformed data. ^¤^ Denotes nonparametric ANOVA. ^#^ Denotes truncated data. Different superscript letters denotes differences between groups. BH2: Dihydrobiopterin, BH4: Tetrahydrobiopterin, Ctrl: Control animals, Def: Vitamin C deficient animals, DHA: Dehydroascorbate, GSH: Glutathione, GSSG: glutathione disulfide, MDA: Malondialdehyde (MDA), NS: Not significant, Sev_def: Severely vitamin C deficient animals, SOD: Superoxide dismutase, VitC: Vitamin C. Tissue levels are shown as amount per gram tissue.

**Table 3:** Synaptic plasticity markers in the frontal cortex, hippocampus and striatum.

	Group/Marker	Ctrl	Def	Sev_Def	One-Way ANOVA
Frontal cortex	*p*-synapsin 1	0.64 ± 0.20	0.54 ± 0.15	0.52 ± 0.15	NS
	synapsin 1	1.41 ± 0.41	1.66 ± 0.80	1.36 ± 0.51	NS
	*p*-synapsin 1/synapsin 1	0.47 ± 0.11	0.41 ± 0.26	0.47 ± 0.29	NS
	*p*-CAMKII	0.08 ± 0.05	0.05 ± 0.03	0.06 ± 0.03	NS
	CAMKII	0.39 ± 0.07	0.38 ± 0.09	0.35 ± 0.05	NS
	*p*-CAMKII/CAMKII	0.23 ± 0.13	0.14 ± 0.08	0.18 ± 0.09	NS
Hippocampus	*p*-synapsin 1	1.24 ± 0.54	1.19 ± 0.74	1.11 ± 0.48	NS
	synapsin 1	1.74 ± 0.46	1.55 ± 0.41	1.51 ± 0.48	NS
	*p*-synapsin 1/synapsin 1	0.71 ± 0.26	0.77 ± 0.39	0.80 ± 0.43	NS
	*p*-CAMKII	0.21 ± 0.12	0.17 ± 0.06	0.16 ± 0.05	NS
	CAMKII	0.31 ± 0.09	0.33 ± 0.09	0.26 ± 0.09	NS
	*p*-CAMKII/CAMKII	0.71 ± 0.40	0.56 ± 0.27	0.63 ± 0.20	NS
Striatum	*p*-synapsin 1	0.42 ± 0.09	0.41 ± 0.13	0.38 ± 0.10	NS
	synapsin 1	2.60 ± 0.92	2.23 ± 0.73	2.33 ± 0.37	NS
	*p*-synapsin 1/synapsin 1	0.18 ± 0.05	0.20 ± 0.11	0.17 ± 0.05	NS
	*p*-CAMKII	ND	ND	ND	-
	CAMKII	0.06 ± 0.04	0.06 ± 0.04	0.04 ± 0.04	NS
	*p*-CAMKII/CAMKII	-	-	-	-

All values are in arbitrary units. No differences between groups were detected in any of the markers, in any of the brain areas. One-way ANOVA with Tukey’s multiple comparison test, *n* = 10, mean ± SD. NS: Not significant. ND: Not detectable. -: Not determined. CAMKII: Ca^2+^-calmodulin-dependent kinase II, Ctrl: Control animals, Def: Vitamin C deficient animals, *p*-CAMKII: phosphorylated Ca^2+^-calmodulin-dependent kinase II, *p*-synapsin 1: phosphorylated synapsin, Sev_def: Severely vitamin C deficient animals.
